# Real-world efficacy and tolerability of ixazomib-based combination therapies in advanced multiple myeloma and other plasma cell neoplasms

**DOI:** 10.1177/20406207261421841

**Published:** 2026-02-28

**Authors:** Xiang Zhou, Julia Mersi, Christine Riedhammer, Maximilian J. Steinhardt, Max Bittrich, Stefan Knop, Hermann Einsele, Leo Rasche, Klaus Martin Kortüm, Johannes M. Waldschmidt

**Affiliations:** Department of Internal Medicine II, University Hospital of Würzburg, Würzburg, Germany; Department of Internal Medicine II, University Hospital of Würzburg, Würzburg, Germany; Department of Internal Medicine II, University Hospital of Würzburg, Würzburg, Germany; Department of Internal Medicine II, University Hospital of Würzburg, Würzburg, Germany; Department of Internal Medicine II, University Hospital of Würzburg, Würzburg, Germany; Department of Internal Medicine 5, Klinikum Nürnberg Nord, Nürnberg, Germany; Department of Internal Medicine II, University Hospital of Würzburg, Würzburg, Germany; Department of Internal Medicine II, University Hospital of Würzburg, Würzburg, Germany; Mildred Scheel Early Career Center, Würzburg, Germany; Department of Internal Medicine II, University Hospital of Würzburg, Würzburg, Germany; Department of Hematology and Oncology, University Hospital Würzburg, Oberdürrbacher Street 6, Würzburg 97080, Germany; Department of Internal Medicine II, University Hospital of Würzburg, Würzburg, Germany

**Keywords:** ixazomib, multiple myeloma, proteasome inhibitor, relapsed/refractory

## Abstract

**Background:**

Ixazomib is an oral proteasome inhibitor for relapsed/refractory multiple myeloma (RRMM). Our study aimed to analyze the efficacy and tolerability of ixazomib-based combination therapies.

**Methods:**

We performed a single-center retrospective analysis of 126 patients with RRMM and other plasma cell neoplasms.

**Results:**

The median age was 65 years, with a median of two prior therapy lines, and 16.7% were triple-class refractory. The overall response rate (ORR) was 52.5%; triple-class refractory patients had a significantly lower ORR than non-refractory controls (10.5% vs 60.2%, *p* < 0.001). After a median follow-up of 27.0 months, the median progression-free survival (PFS) was 7.9 months (95% CI: 6.9–11.0), and the median overall survival (OS) was 84.1 months (95% CI: 68.1–not reached). In multivariate analysis, a glomerular filtration rate of ⩽70 ml/min/1.73 m^2^ was linked to worse PFS (hazard ratio (HR): 2.11, *p* = 0.008) and OS (HR: 7.27, *p* = 0.003). Furthermore, triple-class refractory patients showed a trend toward inferior PFS (HR: 3.76, *p* = 0.05) and significantly worse OS (HR: 20.46, *p* = 0.002) compared to non-refractory patients. Grade ⩾3 hematologic adverse events occurred in 15.1% patients, while the most common non-hematologic adverse events were fatigue (5.6%) and peripheral neuropathy (26.2%), with the majority classified as grade 1–2.

**Conclusion:**

Altogether, ixazomib regimens are potent in RRMM but are less effective in heavily pretreated patients and those with renal impairment, suggesting earlier use may yield greater benefits.

## Introduction

The inhibition of the proteasome represents a major strategy in the therapy of multiple myeloma (MM).^1^ Currently, the three proteasome inhibitors (PIs) bortezomib, carfilzomib and ixazomib are commercially available for the treatment of newly diagnosed (ND) and/or relapsed/refractory (RR) MM, and are already integrated into combination regimens for clinical routine.^2–10^ Ixazomib is the first oral PI, which reversibly inhibits the β5 subunit (chymotrypsin-like) of the 20S proteasome.^11^ In the phase III TOURMALINE-MM1 trial, the addition of ixazomib to the combination of lenalidomide and dexamethasone significantly increased the overall response rate (ORR) and improved the progression-free survival (PFS) in RRMM (median PFS: 20.6 months in ixazomib vs 14.7 months in placebo group; hazard ratio (HR): 0.74, *p* = 0.01).^12^ Based on these results, the combination of Ixa-Rd (ixazomib, lenalidomide, and dexamethasone) was approved for the treatment of RRMM patients with ⩾1 prior therapy in 2015.^11^ Other combination therapies, for example, Ixa-Td (ixazomib, thalidomide, and dexamethasone) and Ixa-Pd (ixazomib, pomalidomide, and dexamethasone), provided similarly encouraging efficacy data in clinical trials.^13,14^ Moreover, Ixa-Rd was evaluated in transplant-ineligible NDMM patients in the TOURMALINE-MM2 trial and led to a clinically meaningful PFS benefit of 13.5 months.^15^ Furthermore, in the TOURMALINE-MM3 study, ixazomib maintenance therapy significantly prolonged PFS in NDMM patients after autologous stem cell transplant (median PFS: 26.5 months in ixazomib vs 21.3 months in placebo group; HR: 0.72, *p* = 0.0023).^16^ Notably, ixazomib, as an oral medication, holds the potential to significantly enhance patients’ quality of life, with “all-oral” regimens like Ixa-Rd being particularly advantageous for elderly patients. In addition, as demonstrated by pooled analysis from multiple clinical trials, ixazomib results in much lower rates of peripheral neuropathy (PN; grade 3: <3%) than bortezomib (grade 3: median 8.1%).^17^ At present, real-world experience with other ixazomib-containing therapies is still limited, especially for combinations other than Ixa-Rd. The optimal positioning of ixazomib in the treatment sequence of RRMM patients, however, still remains to be clarified. Here, we provide a single-center analysis on the real-world efficacy and safety of ixazomib-containing therapies for RRMM.

## Methods

### Patient characteristics

We conducted a single-center retrospective study of 126 patients with RRMM and other plasma cell neoplasms, who were treated with ixazomib-containing therapies between March 2016 and January 2024. All procedures adhered to the Declaration of Helsinki and Good Clinical Practice guidelines. Informed consent was obtained from all patients included in the analysis. Given the retrospective design of this study, the Ethics Committee of the University of Würzburg waived the need for additional approval. Collected data included demographics, cytogenetics, prior treatments, therapy regimens, responses, adverse events, and survival outcomes. High-risk cytogenetics were determined by fluorescence in situ hybridization and defined as the presence of at least one of the following abnormalities: t(4;14), +1q21, or del17p.^18^ Extramedullary disease (EMD) was assessed by positron emission tomography/computed tomography or whole-body diffusion-weighted magnetic resonance imaging.

### Treatment schedules and dosing

Patients received ixazomib orally on days 1, 8, and 15 of a 4-week cycle, with cycles repeated on day 29. In regimens containing immunomodulatory drugs (IMiDs) such as Ixa-Rd (lenalidomide–dexamethasone), Ixa-Pd (pomalidomide–dexamethasone), and Ixa-Td (thalidomide–dexamethasone), IMiDs were administered orally once daily on days 1–21. Drug dosing was determined at the discretion of the treating physician. Alternative IMiD-free regimens in this study included Ixa-Ed (ixazomib–elotuzumab–dexamethasone), Ixa-Dd (ixazomib–daratumumab–dexamethasone), Ixa-Cd (ixazomib–cyclophosphamide–dexamethasone), or Ixa-d (ixazomib–dexamethasone). Elotuzumab and daratumumab were administered intravenously or subcutaneously as per prescribing information. Cyclophosphamide dosing is detailed in Table 1. Except for ixazomib monotherapy, dexamethasone was given orally or intravenously on ixazomib treatment days. Therapy continued until disease progression or intolerable toxicity. Patients received prophylaxis against *Pneumocystis jirovecii* (e.g., co-trimoxazole 960 mg orally every other day) and herpes viruses (e.g., acyclovir 400 mg orally twice daily). Thrombosis prophylaxis was provided for IMiD regimens (e.g., enoxaparin 40 mg subcutaneously once daily or aspirin 100 mg orally once daily). Supportive therapies, including hematopoietic growth factors and blood transfusions, were administered per institutional practice and international guidelines.^19^

**Table 1.** Therapy regimens.

Parameter	N/(%)
Regimens	
Ixazomib monotherapy maintenance	7 (5.5)
Ixa-d	16 (12.7)
Ixa-Rd	59 (46.8)
Ixa-Pd	25 (19.9)
Ixa-Td	12 (9.5)
Ixa-Cd	4 (3.2)
Ixa-Dd	2 (1.6)
Ixa-Ed	1 (0.8)
Starting dosing of ixazomib	
4 mg	74 (58.7)
3 mg	21 (16.7)
2.3 mg	2 (1.6)
NA	29 (23.0)
Dosing of drugs other than ixazomib or dexamethasone	
Cyclophosphamide (Ixa-Cd: *n* = 4)	
50 mg QD	1 (25)
300 mg/m^2^ QW	1 (25)
1500 mg absolute QW	1 (25)
NA	1 (25)
Lenalidomide (Ixa-Rd: *n* = 59)	
25 mg QD	13 (22)
15 mg QD	9 (15)
10 mg QD	9 (15)
5 mg QD	4 (7)
NA	24 (41)
Pomalidomide (Ixa-Pd: *n* = 25)	
4 mg QD	18 (72)
3 mg QD	2 (8)
2 mg QD	3 (12)
NA	2 (8)
Thalidomide (Ixa-Td: *n* = 12)	
100 mg QD	9 (75)
100 mg QOD	1 (8)
50 mg QD	2 (17)
Intent of treatment, *n* (%)	
Maintenance therapy	14 (11.1)
Relapse therapy	112 (88.9)

Ixa-Cd, ixazomib–cyclophosphamide–dexamethasone; Ixa-d, ixazomib–dexamethasone; Ixa-Dd, ixazomib–daratumumab–dexamethasone; Ixa-Ed, ixazomib–elotuzumab–dexamethasone; Ixa-Pd, ixazomib–pomalidomide–dexamethasone; Ixa-Rd, ixazomib–lenalidomide–dexamethasone; Ixa-Td, ixazomib–thalidomide–dexamethasone.

### Response assessment and adverse events

Response to treatment was evaluated using the current International Myeloma Working Group (IMWG) Response Criteria.^20^ Overall survival (OS) was measured from the start of ixazomib-containing therapy to death or last follow-up. PFS was defined as the time from therapy initiation to relapse, progression, or the last follow-up if no relapse or progression occurred. Adverse events were classified according to the Common Terminology Criteria for Adverse Events (CTCAE) Version 5.0.

### Statistical analysis

For descriptive statistics, unless otherwise specified, data were reported as absolute numbers with percentages or medians with ranges. Comparisons between subgroups were conducted using the Mann–Whitney *U* test for continuous variables and Fisher’s exact test for categorical data. Univariate survival analysis was performed using the Kaplan–Meier method, while multivariate analysis employed the Cox regression model. All analyses were conducted using R (version 4.3.2), with a *p* value of less than 0.05 considered statistically significant. Source data from this study will be made available by the corresponding author upon reasonable request.

## Results

### Patient characteristics

Most patients were male (*n* = 72/126, 57.1%), with a median age of 65 (range: 37–86) years at the start of ixazomib treatment. The median time from MM diagnosis to ixazomib initiation was 4.4 (range: 3.1–7.2) years. High-risk cytogenetics were identified in 61 (48.4%) patients, and 15 (11.9%) had EMD at the start of therapy. The median glomerular filtration rate (GFR) at initiation was 70 (range: 8–141) ml/min/1.73 m^2^. Patients had a median of two prior therapy lines (range: 1–13), with 35 (27.8%) patients receiving ixazomib in second line. Among them, 49 (38.9%), 41 (32.5%), and 29 (23.0%) patients were refractory to at least one IMiD, PI, and anti-CD38 antibody, respectively, while 21 (16.7%) were refractory to all three (triple-class refractory). In addition, 100 (79.4%) patients were exposed to autologous and 12 (9.5%) had received allogeneic stem cell transplantation. Three patients received upfront autologous-allogeneic tandem transplant in the first line of therapy, with only one patient showing high-risk cytogenetics (isolated gain 1q21). Two (1.6%) patients had been treated with the B-cell maturation antigen (BCMA)-targeted antibody–drug conjugate belantamab–mafodotin, and three (2.4%) were treated with the bispecific T-cell engager AMG420. Notably, our cohort included a minor proportion of patients with co-existing conditions: nine (7.1%) with AL amyloidosis, two (1.6%) with POEMS syndrome, and two (1.6%) with monoclonal gammopathy of clinical significance accompanied by neuropathy. Patient characteristics are summarized in Table 2.

**Table 2.** Patient characteristics and response to treatment.

Parameter	N(%)
Patients, *n*	126
Gender, *n* (%)	
Male	72 (57.1)
Female	54 (42.9)
Age at MM diagnosis, median (range), years	59 (32–84)
Age at ixazomib start, median (range), years	65 (37–86)
Time between MM diagnosis and ixazomib start, median (range), months	53 (1–280)
MM-associated diseases, *n* (%)	
AL amyloidosis	9 (7.1)
POEMS syndrome	2 (1.6)
MGCS (neuropathy)	2 (1.6)
Subtype, *n* (%)	
IgG	66 (52.4)
IgA	36 (28.6)
LC	24 (19.0)
Cytogenetics, *n* (%)	
High risk*	61 (48.4)
Standard risk	44 (34.9)
NA	21 (16.7)
Prior lines of therapy, *n* (%)	
1	35 (27.8)
2–3	55 (43.7)
⩾4	36 (28.5)
Extramedullary disease, *n* (%)	
Yes	15 (11.9)
No	111 (88.1)
Prior treatment, *n* (%)	Exposed/Refractory
IMiDs	100 (79.4)/49 (38.9)
Lenalidomide	100 (79.4)/45 (35.7)
Pomalidomide	30 (23.8)/22 (17.5)
PIs	119 (94.4)/41 (32.5)
Bortezomib	116 (92.1)/33 (26.2)
Carfilzomib	37 (29.4)/16 (12.7)
Monoclonal antibodies	
Daratumumab	48 (38.1)/29 (23.0)
Elotuzumab	13 (10.3)/11(8.7)
Triple-class refractory**	21 (16.7)
Prior SCT	
Autologous SCT	100 (79.4)
Allogenic SCT	12 (9.5)
Prior BCMA-directed novel immunotherapy	
ADC	2 (1.6)
BITE	3 (2.4)
Best response to ixazomib (data evaluable: *n* = 122), *n* (%)	
CR	11 (9.0)
VGPR	22 (18.0)
PR	31(25.5)
MR	33 (27.0)
PD	25 (20.5)
Best response grouped by regimens, *n* (%)^$^	
Ixa-Rd (data evaluable: *n* = 57)	
CR	5 (8.8)
VGPR	8 (14.0)
PR	15 (26.3)
MR	15 (26.3)
PD	14 (24.6)
Ixa-Pd (data evaluable: *n* = 23)	
CR	2 (8.7)
VGPR	3 (13.1)
PR	8 (34.8)
MR	5 (21.7)
PD	5 (21.7)

* Presence of at least one of the following: del(17p), +1q21, t(4;14).

** Refractory to PI, IMiD, and daratumumab.

$ Only regimens with >20 patients are presented.

ADC, antibody–drug conjugate; BCMA, B-cell maturation antigen; BITE, bispecific T-cell engager; CR, complete response; IMiDs, immunomodulatory drugs; Ixa-Pd, ixazomib–pomalidomide–dexamethasone; Ixa-Rd, ixazomib–lenalidomide–dexamethasone; LC, light chain; MGCS, monoclonal gammopathy of clinical significance; MM, multiple myeloma; MR, minor response; NA, not available; PD, progressive disease; PIs, proteasome inhibitors; PR, partial response; SCT, stem cell transplant; VGPR, very good partial response.

### Regimens and dosing

In 112 (88.9%) patients, ixazomib was administered as a treatment for MM relapse. Most patients (*n* = 74, 58.7%) started ixazomib at a 4 mg QW dose and received either doublet therapy (Ixa-d, *n* = 15, 13.4%) or triplet combinations (*n* = 97, 86.6%). Among these, 91 (81.3%) patients were treated with IMiD-based regimens: Ixa-Rd (*n* = 55, 46.8%), Ixa-Pd (*n* = 24, 19.9%), or Ixa-Td (*n* = 12, 9.5%), while Ixa-Cd and Ixa-Dd were less frequently used. The remaining 14 patients (11.1%) received ixazomib as part of relapse therapy (secondary maintenance), with ixazomib monotherapy (*n* = 7) and Ixa-Rd (*n* = 4) having been the most commonly utilized options. Therapy regimens and doses are detailed in Table 1.

### Response to therapy

Among the 122 patients (96.8%) with available response data, the ORR was 52.5%, with 11 (9.0%), 22 (18.0%), and 31 (25.5%) patients achieving complete response (CR), very good partial response (VGPR), and partial response (PR), respectively. In addition, 33 (27.0%) patients achieved a minor response (MR), resulting in a clinical benefit rate (CBR) of 79.5%. Patients receiving ixazomib in the second line demonstrated a significantly higher ORR compared to those receiving ixazomib at later lines (77.1% vs 42.5%, *p* = 0.0006). Furthermore, patients refractory to other PIs in prior therapies showed a significantly lower ORR than their counterparts (28.9% vs 61.1%, *p* = 0.0008). Triple-class refractory patients exhibited a notably inferior ORR (10.5%, *p* < 0.0001). In the entire cohort, the presence of EMD, high-risk cytogenetics, and age ⩾80 years did not correlate with lower ORR (Figure S1).

The ORR in Ixa-Rd-treated patients (*n* = 57/59 assessable) was 49.1%, with 5 (8.8%), 8 (14.0%), and 15 (26.3%) achieving a CR, VGPR, and PR, respectively. In addition, 15 (26.3%) achieved a MR, yielding a CBR of 75.4%. In the Ixa-Rd subgroup, patients with more than one prior line of therapy were confirmed to have a lower ORR compared to those with only one prior line of treatment (37.5% vs 76.5%, *p* = 0.0096). Patients refractory to other PIs (23.8% vs 63.9%, *p* = 0.0056) or triple-class refractory (8.3% vs 60.0%, *p* = 0.0011) also displayed significantly lower ORRs. Moreover, high-risk cytogenetics were associated with lower ORR compared to standard cytogenetics in the Ixa-Rd subgroup (35.7% vs 70.0%, *p* = 0.039; Figure S2). Response data grouped by regimen are presented in Table 2. Due to the limited number of cases, we did not analyze response data for ixazomib combinations with ⩽20 patients.

### Survival analyses

**Figure 1.**
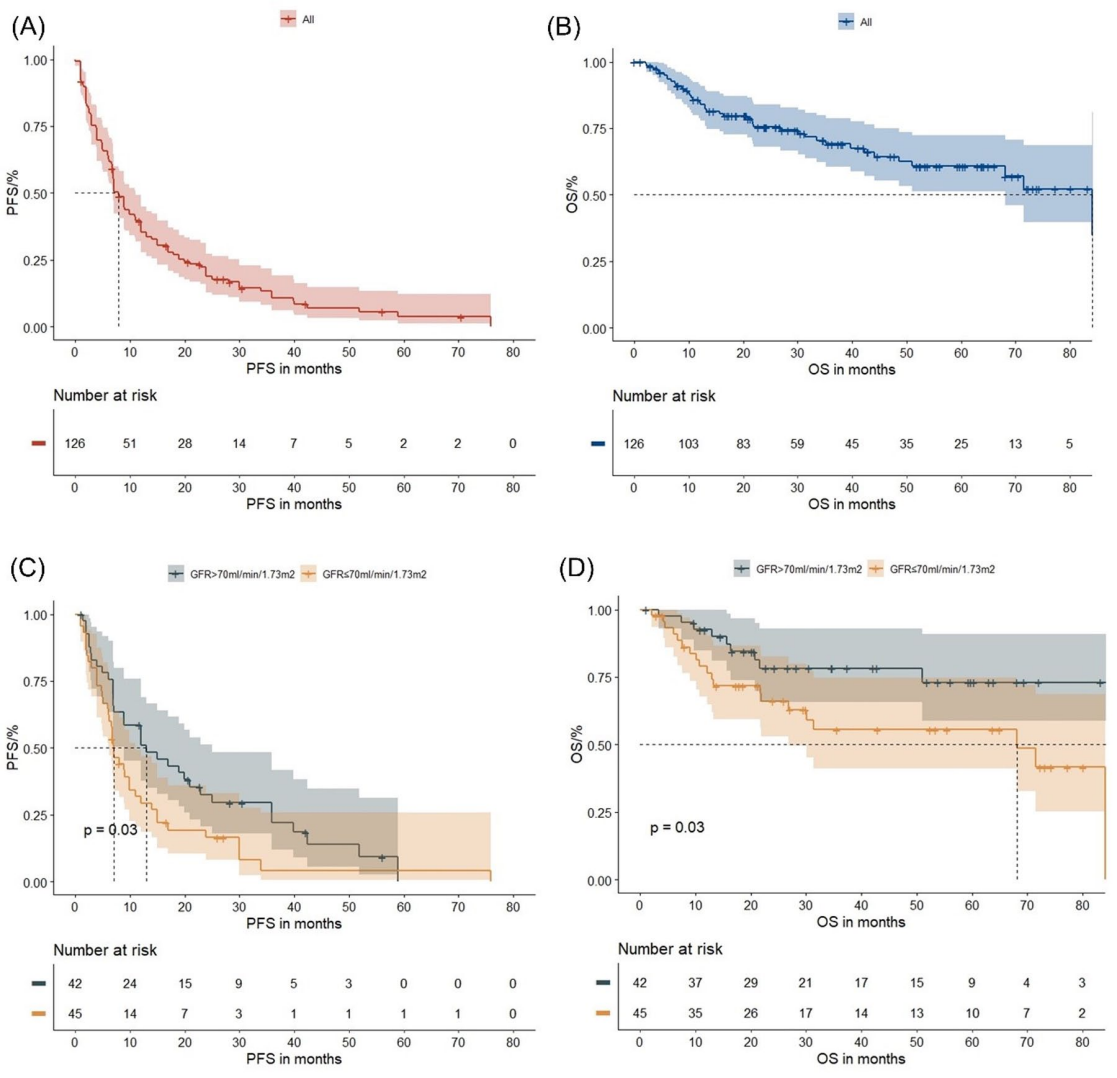
Survival outcomes and renal function in the entire group: The figures illustrate the PFS and OS in the entire group (a, b) and in patients with GFR >70 versus ⩽70 ml/min/1.73 m^2^ (c, d). Log-rank *p* values are provided. GFR, glomerular filtration rate; OS, overall survival; PFS, progression-free survival.

At a median follow-up of 27.0 months, the PFS and OS for the entire cohort were 7.9 months (95% CI: 6.9–11.0) and 84.1 months (95% CI: 68.1–not reached), respectively (Figure 1(a) and (b)). No significant differences in PFS or OS were observed between patients receiving IMiD-containing versus IMiD-free combinations (both *p* > 0.05). In our study, 12 and 84 patients were censored due to loss of follow-up in terms of PFS and OS, respectively. Two patients were censored due to non-MM-related deaths.

As expected, clinical responders (PR or better) had significantly longer PFS (18.9 months vs 3.5 months, *p* < 0.0001) and OS (84.1 months vs 30.1 months, *p* < 0.0001) as compared to non-responders (Figure S3(A) and (B)). Patients with EMD showed a trend toward shorter PFS (3.9 months vs 8.0 months, *p* = 0.068) and OS (42.3 months vs 84.1 months, *p* = 0.10) compared to those without (Figure S4(A) and (B)). Elderly patients (⩾80 years) had similar PFS (*p* = 0.59) but expectedly shorter OS (26.1 months vs 84.1 months, *p* = 0.039) compared to younger patients (Figure S4(C) and (D)). High-risk cytogenetics did not significantly affect PFS (*p* = 0.24) or OS (*p* = 0.25; Figure S4(E) and (F)). However, patients with a GFR ⩽70 ml/min/1.73 m² had shorter PFS (7.0 months vs 12.9 months, *p* = 0.03) and OS (68.1 months vs 86.8 months, *p* = 0.03) compared to those with a GFR >70 ml/min/1.73 m^2^ (Figure 1(c) and (d)).

**Figure 2.**
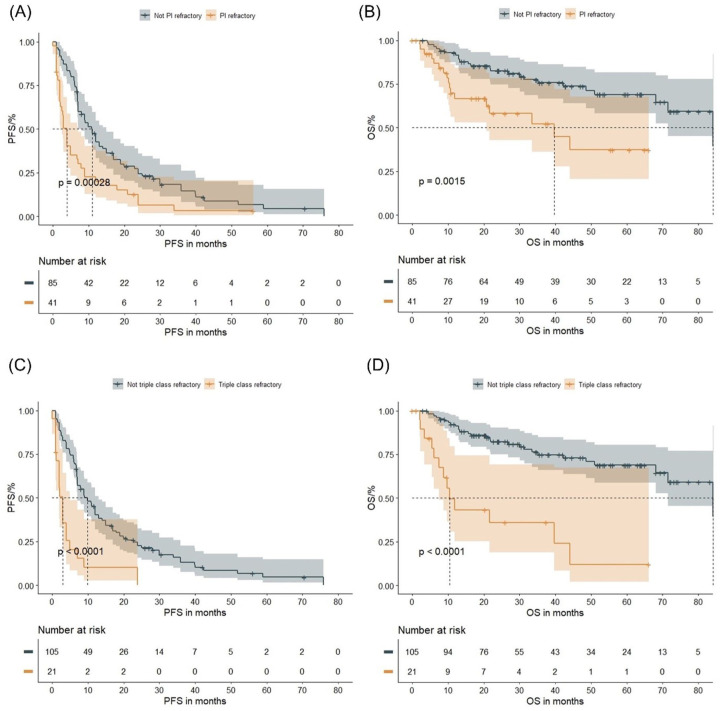
Survival outcomes and pretreatment in the entire group: The figures demonstrate the PFS and OS in PI-refractory versus non-PI-refractory patients (a, b), and in patients being triple-class refractory versus not triple-class refractory (c, d). Log-rank *p* values are provided. OS, overall survival; PFS, progression-free survival; PI, proteasome inhibitor.

Patients with >1 prior line of therapy had significantly shorter PFS (6.9 months vs 17.0 months, *p* = 0.00018) and OS (68.1 months vs 84.1 months, *p* = 0.03) as compared to those with only one prior line of therapy (Figure S3(C) and (D)). PI-refractory patients showed inferior PFS (3.9 months vs 10.9 months, *p* = 0.00028) and OS (39.7 months vs 84.1 months, *p* = 0.0015) as compared to non-refractory patients (Figure 2(a) and (b)). In addition, triple-class refractory patients had shorter PFS (3.0 months vs 9.9 months, *p* < 0.0001) and OS (10.4 months vs 84.1 months, *p* < 0.0001) as compared to others (Figure 2(c) and (d)).

**Figure 3.**
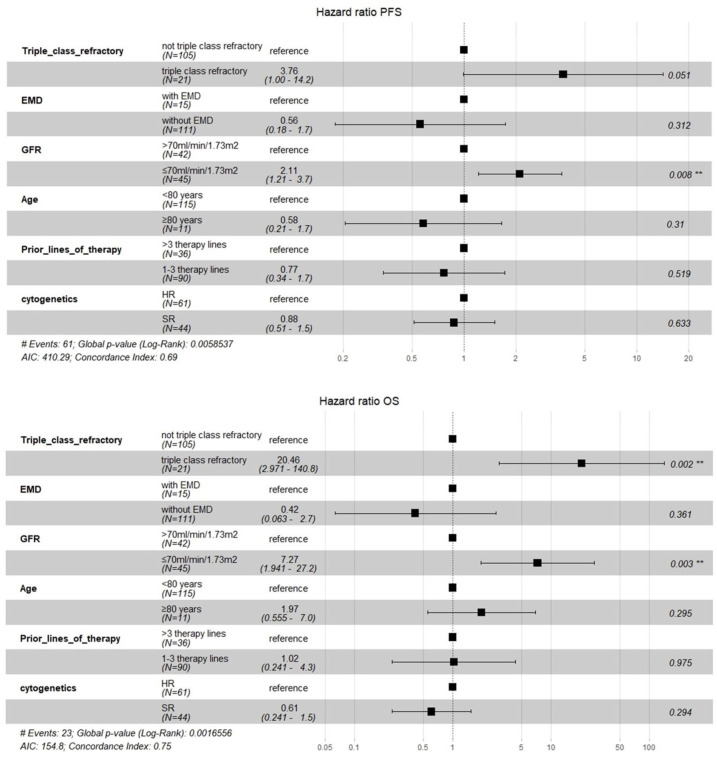
Multivariate survival analysis in the entire group: This figure displays the prognostic roles of refractoriness, renal function, EMD, cytogenetics, and age of patients in the entire cohort. Hazard ratio, 95% confidence interval, and *p* values are presented. EMD, extramedullary disease; GFR, glomerular filtration rate; HR, high risk; OS, overall survival; PFS, progression-free survival; SR, standard risk.

Multivariate survival analysis identified a GFR ⩽70 ml/min/1.73 m^2^ as an independent prognostic risk factor for worse PFS (HR: 2.11, 95% CI: 1.21–3.70, *p* = 0.008) and OS (HR: 7.27, 95% CI: 1.94–27.20, *p* = 0.003). Patients who were triple-class refractory displayed a non-significant trend toward worse PFS (HR: 3.76, 95% CI: 1.00–14.20, *p* = 0.051) and significantly inferior OS (HR: 20.46, 95% CI: 2.97–140.80, *p* = 0.002; Figure 3).

**Figure 4.**
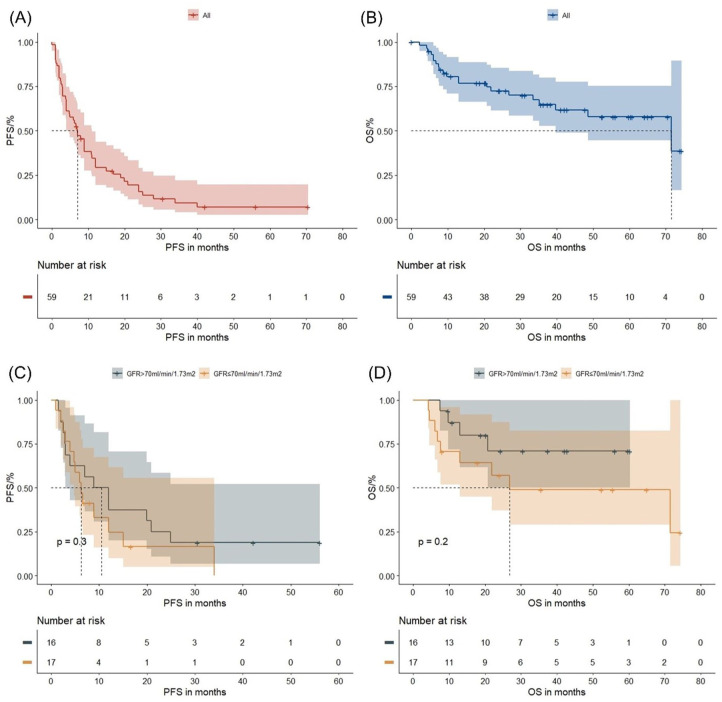
Survival outcomes and renal function in the subgroup Ixa-Rd: The figures illustrate the PFS and OS in the entire group (a, b), and in patients with GFR >70 versus ⩽70 ml/min/1.73 m^2^ (c, d). Log-rank *p* values are provided. GFR, glomerular filtration rate; Ixa-Rd, ixazomib–lenalidomide–dexamethasone; OS, overall survival; PFS, progression-free survival.

**Figure 5.**
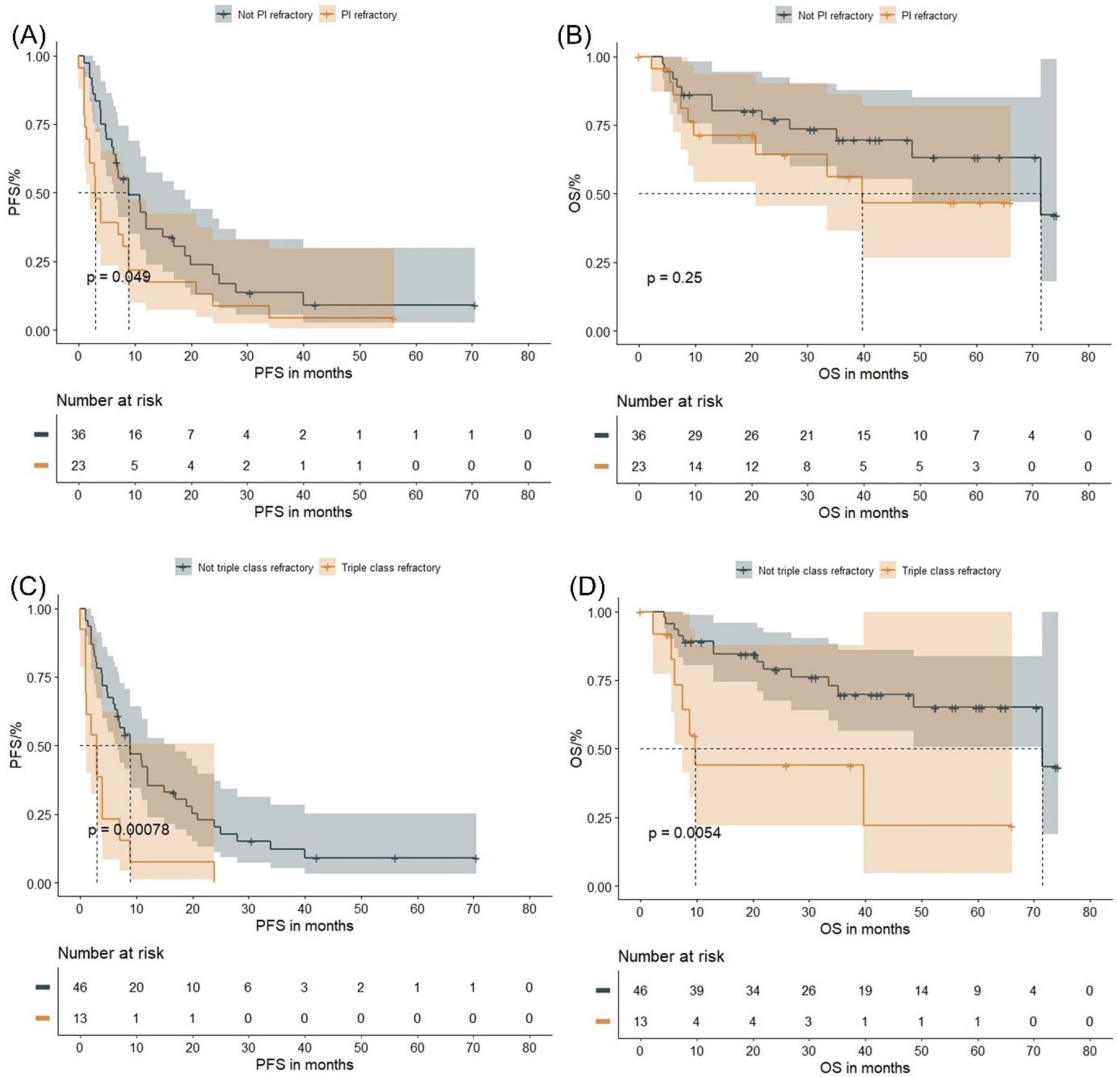
Survival outcomes and pretreatment in the subgroup Ixa-Rd: The figure demonstrates the PFS and OS in PI-refractory versus non-PI-refractory patients (a, b), and in patients being triple-class refractory versus not triple-class refractory (c, d). Log-rank *p* values are provided. Ixa-Rd, ixazomib–lenalidomide–dexamethasone; OS, overall survival; PFS, progression-free survival; PI, proteasome inhibitor.

In the Ixa-Rd subgroup, median PFS and OS were 7.0 months (95% CI: 4.0–11.0) and 71.5 months (95% CI: 39.7–not reached), respectively (Figure 4(a) and (b)). Responders to Ixa-Rd had significantly longer PFS (19.9 months vs 3.0 months, *p* < 0.0001) and OS (26.9 months vs not reached, *p* = 0.00015) compared to non-responders (Figure S5(A) and (B)). Surprisingly, a GFR ⩽70 ml/min/1.73 m^2^ did not correlate with shorter PFS or OS in the Ixa-Rd subgroup (both *p* > 0.05), probably due to the low number of patients (Figure 4(c) and (d)). Patients with ⩽3 prior therapy lines exhibited significantly longer PFS (10.9 months vs 3.0 months, *p* = 0.009) and OS (71.5 months vs 39.7 months, *p* = 0.04) compared to those with >3 lines (Figure S5(C) and (D)). Furthermore, PI-refractory patients had shorter PFS (3.0 months vs 8.9 months, *p* = 0.049) compared to non-refractory patients (Figure 5(a) and (b)). Triple-class refractory patients demonstrated significantly shorter PFS (3.0 months vs 8.9 months, *p* = 0.00078) and OS (9.8 months vs 71.5 months, *p* = 0.0054) compared to other patients (Figure 5(c) and (d)). EMD was not associated with poorer survival outcomes (both *p* > 0.05, Figure S6(A) and (B)). Elderly patients over 80 years had similar PFS but shorter OS (17.3 months vs not reached, *p* = 0.0075) compared to younger patients (Figure S6(C) and (D)). High-risk cytogenetics indicated shorter PFS (4.0 months vs 15.0 months, *p* = 0.0053) and a trend toward shorter OS (39.7 months vs not reached, *p* = 0.069) compared to standard-risk cytogenetics (Figure S6(E) and (F)). Due to a limited number of cases, we did not perform multivariate analysis in the Ixa-Rd subgroup. In addition, we did not observe any differences in PFS or OS between the subgroups IMiD-containing versus IMiD-free combinations (Figure S7(A) and (B)). Likewise, the subgroups Ixa-Rd, Ixa-Pd, and Ixa-Td showed no significant difference in terms of PFS or OS (Figure S7(C) and (D)). Moreover, the presence of MM-associated AL amyloidosis did not influence the survival outcome of the patients (Figure S8(A) and (B)). Furthermore, the patients treated with ixazomib-containing maintenance showed longer PFS (19.9 months vs 7.0 months, *p* = 0.047) and OS (not reached vs 71.5 months, *p* = 0.043) compared to the patients who received ixazomib due to relapse or refractory disease. However, the results only marginally reached statistical significance (Figure S9(A) and (B)).

### Adverse events

Overall, adverse events in this real-world cohort were mild and in line with previous reports on ixazomib toxicity.^2–4^ Grade ⩾2 hematologic adverse events were reported in 40 patients (31.7%), including grade ⩾3 in 19 (15.1%) patients (7.9% anemia, 5.5% leukopenia, 4.8% thrombocytopenia). Grade 4 thrombocytopenia was observed in three patients (2.4%), all of whom were treated with IMiD-containing combinations (Ixa-Rd: *n* = 2, Ixa-Pd: *n* = 1). An overview of hematologic adverse events is provided in Table 3.

**Table 3.** Adverse events.

Events	Any grade ⩾2	Grade 3	Grade 4
Hematologic events, *n* (%)			
Anemia	25 (19.8)	10 (7.9)	3 (2.4)
White blood cells decreased	20 (15.9)	7 (5.5)	
Neutrophil count decreased	18 (14.3)	10 (7.9)	
Platelet count decreased	16 (12.7)	3 (2.4)	
Non-hematologic events, *n* (%)			
Fatigue	7 (5.5)		
Peripheral polyneuropathy	7 (5.5)		
Respiratory infection	3 (2.4)		
Pneumonia	2 (1.6)	2 (1.6)	
Abdominal distension	2 (1.6)	1 (0.8)	
Thromboembolic event	2 (1.6)		
Rash	2 (1.6)		
Bleeding	1 (0.8)		
Nausea	1 (0.8)		
Hearing loss	1 (0.8)		

**Figure 6.**
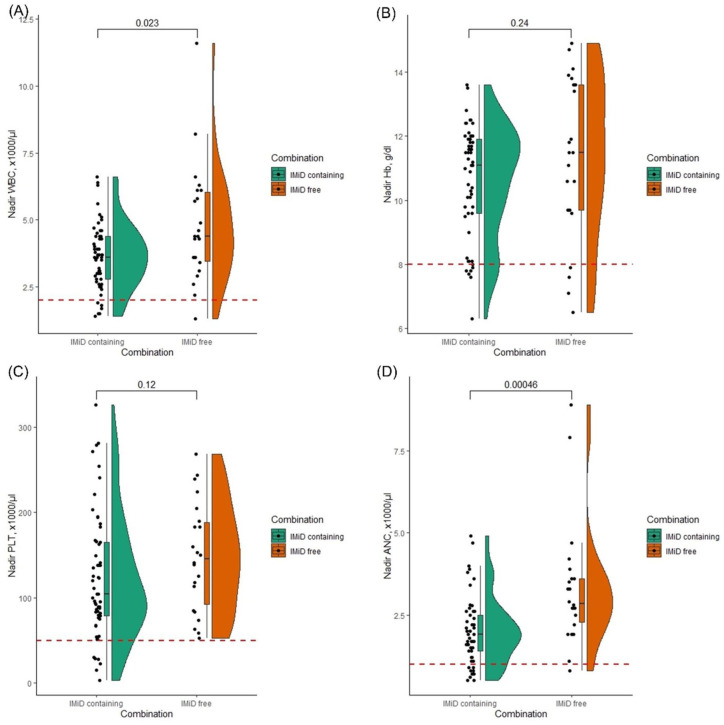
Nadir blood count in IMiD-containing versus IMiD-free regimens: The figure displays the nadir blood counts, that is, WBC (a), Hb (b), PLT (c), and ANC (d), in patients treated with IMiD-containing versus IMiD-free ixazomib combinations. The dashed red lines show the cut-off values for grade 3 hematologic toxicities according to the CTCAE classification version 5.0. ANC, absolute neutrophil count; CTCAE, Common Terminology Criteria for Adverse Events; Hb, hemoglobin; IMiD, immunomodulatory drug; PLT, platelet count; WBC, white blood cell count.

To note, patients treated with IMiD combinations had significantly lower nadir white blood cell (WBC) counts (median: 3.6 vs 4.4 ×1000/µl, *p* = 0.023) and nadir absolute neutrophil counts (ANCs; median: 1.9 vs 2.8 ×1000/µl, *p* = 0.00046) than those treated with other combinations, indicating that IMiDs were the major contributor to leukopenia and neutropenia (Figure 6).

**Figure 7.**
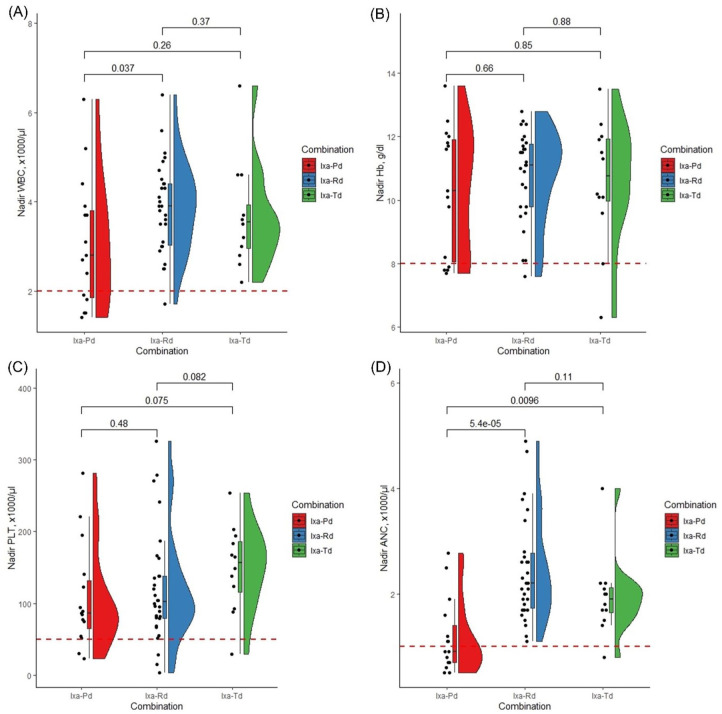
Nadir blood counts in different IMiD-containing regimens: The figure displays the nadir blood counts, that is, WBC (a), Hb (b), PLT (c), and ANC (d), in patients treated with different IMiD-containing ixazomib combinations. The dashed red lines show the cut-off values for grade 3 hematotoxicities according to the CTCAE classification version 5.0. ANC, absolute neutrophil count; CTCAE, Common Terminology Criteria for Adverse Events; Hb, hemoglobin; IMiD, immunomodulatory drug; Ixa-Pd, ixazomib–pomalidomide–dexamethasone; Ixa-Rd, ixazomib–lenalidomide–dexamethasone; Ixa-Td, ixazomib-thalidomide–dexamethasone; PLT, platelet count; WBC, white blood cell.

Among patients receiving IMiD regimens, those on Ixa-Pd had significantly lower nadir ANC compared to Ixa-Rd (median: 0.9 vs 2.2 ×1000/µl, *p* < 0.0001) and Ixa-Td (median: 0.9 vs 1.9 ×1000/µl, *p* = 0.0096; Figure 7), indicating that pomalidomide particularly contributes to hematologic adverse events.

Non-hematologic adverse events (any grade) were documented in 46 patients (36.5%), with fatigue (*n* = 7, 5.5%, all grade 2) and peripheral neuropathy (PN, grade 1: *n* = 27, 21.4%; grade 2: *n* = 6, 4.8%) being the most common. Preexisting PN grade 1 was reported in 28 (22.2%) patients, including 1 patient with prior thalidomide and 25 patients with prior bortezomib exposure. PN symptoms resolved in three patients, all with prior bortezomib treatment, indicating a likely association with earlier PI exposure. In 19 patients, PN remained stable, while it worsened in 6 patients following ixazomib therapy. In addition, eight patients without preexisting PN developed new symptoms during treatment (grade 1: *n* = 7, grade 2: *n* = 1). Overall, ixazomib-containing treatments were well tolerated, with the majority of adverse events being grade <3. In our study, ixazomib dose reduction was required in two patients due to fatigue grade 2 (3–2.3 mg QW) and PN grade 2 (4–3 mg QW), respectively.

## Discussion

Real-world experience with ixazomib-containing therapies, especially combinations beyond Ixa-Rd, remains limited in relapsed/refractory multiple myeloma (RRMM).^11^ In this study, we therefore analyzed clinical data from 126 RRMM patients treated with ixazomib-containing regimens.

The ORR in our study was 52.5% for the entire cohort and 49.1% for the Ixa-Rd subgroup, both significantly lower than those reported in the pivotal TOURMALINE-MM1 trial, which led to the approval of Ixa-Rd in RRMM.^12^ Furthermore, other real-world studies on Ixa-Rd have reported ORRs as high as 85%.^21,22^ Notably, most patients in these studies, including TOURMALINE-MM1, had received only 1–3 prior lines of therapy. By contrast, our cohort included a substantial proportion of heavily pretreated patients, with 36 (28.6%) receiving >3 prior therapies, 41 (32.5%) being refractory to other PIs, and 21 (16.7%) being classified as triple-class refractory. Consistently, both in the entire cohort and the Ixa-Rd subgroup, we observed a significantly lower ORR among heavily pretreated patients (those with >3 prior therapies, PI refractory, or triple-class refractory) compared to those with just one prior therapy. In the REMIX study evaluating real-world outcomes of Ixa-Rd, the ORR was 73.1%, but dropped to 54.4% in patients treated in fourth-line or later settings.^23^

To the best of our knowledge, no real-world studies on ixazomib-containing therapies other than Ixa-Rd have been published in RRMM. In our cohort, 25 patients received Ixa-Pd, all of whom had prior exposure to lenalidomide and a PI. The ORR in this subgroup was 56.5%, which is comparable to results from two phase I/II trials (ORRs of 48.0% and 51.7%) investigating Ixa-Pd in RRMM.^14,24^ Overall, ixazomib-containing regimens, including Ixa-Rd and Ixa-Pd, demonstrated efficacy in RRMM, but their response rates diminished in heavily pretreated patients with >3 prior lines of therapy, suggesting that these regimens should be administered earlier in the treatment course.

In terms of survival outcomes, the median PFS for the entire cohort was 7.9 months, similar to the PFS observed in the Ixa-Rd subgroup (7.0 months). This is notably lower than the median PFS reported in the TOURMALINE-MM1 trial (20.6 months) and other real-world Ixa-Rd studies (up to 43 months), likely reflecting the more heavily pretreated nature of our cohort.^12,21,22^ In the Ixa-Pd subgroup, the median PFS was 7.0 months, aligning with phase I/II trial results from Krishnan et al. (8.6 months) and Voorhees et al. (4.4 months), where patients had also been exposed to PIs and were refractory to lenalidomide.^14,24^ Multivariate analysis identified impaired renal function (GFR ⩽70 ml/min/1.73 m^2^) and triple-class refractory disease as negative prognostic factors for both PFS and OS, highlighting the challenges of managing heavily pretreated, multi-refractory patients with renal impairment. In this context, ixazomib-based treatments do not appear to provide a substantial therapeutic advancement.

Overall, the treatment was well tolerated. Grade ⩾3 hematologic adverse events were observed in 19 patients (15.1%). Grade ⩾3 neutropenia and leukopenia were largely attributable to IMiD use, particularly pomalidomide, as evidenced by our finding that nadir WBC and ANCs were significantly lower in IMiD-containing ixazomib regimens compared to IMiD-free regimens. Importantly, the global rate of infection was very low in our study, even in patients treated with Ixa-Pd, most likely due to the universal use of co-trimoxazole prophylaxis. PN was the most common non-hematologic adverse event, occurring in 27 patients (21.4%) with grade 1 and in 6 patients (4.8%) with grade 2. Preexisting PN related to POEMS syndrome, thalidomide, or bortezomib treatment likely contributed to PN development, complicating the assessment of ixazomib’s role in PN. Interestingly, in three bortezomib-pretreated patients, PN symptoms resolved during ixazomib treatment. The underlying mechanisms of PN associated with PIs like bortezomib and ixazomib warrant further investigation.^25,26^ Comparative analyses between different regimens in this study were limited by small subgroup sizes, such as for Ixa-Td, Ixa-Cd, and Ixa-d.

Currently, the optimal positioning of ixazomib within the treatment landscape of MM has not been fully defined. Chimeric antigen-receptor (CAR) T-cell therapy and anti-CD38 antibodies are moving toward the earlier therapy lines, for example, ciltacabtagene autoleucel is now approved by EMA and FDA for MM patients at first relapse. In turn, an increasing number of lenalidomide- or anti-CD38 antibody-refractory patients are expected after first-line treatment. Therefore, PI-based regimens will be an option to bridge to CAR T-cell therapy in the second line. Due to its availability as an oral drug and given its manageable safety profile, ixazomib could be particularly attractive for elderly patients, also in later lines of therapy. In addition, ixazomib may depict another option as maintenance after first-line therapy, potentially also to provide an alternative to long-term lenalidomide and its detrimental effects on secondary primary malignancies, including secondary myelodysplastic neoplasm and acute myeloid leukemia.^27,28^

Our study has several limitations. First, this is a retrospective study based on a relatively small and heterogeneous patient cohort, including a drop-out rate and follow-up, which are intrinsically limited as typically seen in real-world studies. Second, high-risk cytogenetics were defined as per the R2-ISS classification and did not contain *del1p32* and *TP53* mutations, as recently updated in the novel IMWG high-risk definition.^29^ Third, some data, for example, frailty and quality of life, were not routinely recorded in our retrospective dataset, and the results of our study should therefore be interpreted with caution.

## Conclusion

Our real-world experience indicates that ixazomib-containing regimens are effective in RRMM. However, their efficacy decreases in heavily pretreated and multi-refractory patients, supporting the need for ixazomib combinations to be used earlier in treatment. PN remains one of the most common adverse events and warrants further investigation.

## Supplemental Material

sj-docx-1-tah-10.1177_20406207261421841 – Supplemental material for Real-world efficacy and tolerability of ixazomib-based combination therapies in advanced multiple myeloma and other plasma cell neoplasms Supplemental material, sj-docx-1-tah-10.1177_20406207261421841 for Real-world efficacy and tolerability of ixazomib-based combination therapies in advanced multiple myeloma and other plasma cell neoplasms by Xiang Zhou, Julia Mersi, Christine Riedhammer, Maximilian J. Steinhardt, Max Bittrich, Stefan Knop, Hermann Einsele, Leo Rasche, Klaus Martin Kortüm and Johannes M. Waldschmidt in Therapeutic Advances in Hematology

## Declarations

**Ethics approval and consent to participate:** The Ethics Committee of the University of Würzburg waived the need for additional approval of retrospective studies.

**Consent for publication:** Not applicable.

**Availability of data and materials:** The data that support the findings of this study are available from the corresponding author(s) upon reasonable request.
