# Supplementary material for: Real-world efficacy and tolerability of ixazomib-based combination therapies in advanced multiple myeloma and other plasma cell neoplasms
Source: Ther Adv Hematol. 2026 Feb 28;17:20406207261421841. doi: 10.1177/20406207261421841 (PMC12953948; doi:10.1177/20406207261421841)
Supplement: sj-docx-1-tah-10.1177_20406207261421841 – Supplemental material for Real-world efficacy and tolerability of ixazomib-based combination therapies in advanced multiple myeloma and other plasma cell neoplasms [file sj-docx-1-tah-10.1177_20406207261421841.docx]

**Supplementary information:**

**Real-world efficacy and tolerability of ixazomib-based combination therapies in advanced multiple myeloma** **and other plasma cell neoplasms**

Xiang Zhou^1^, Julia Mersi^1^, Christine Riedhammer^1^, Maximilian J. Steinhardt^1^, Max Bittrich^1^, Stefan Knop^2^, Hermann Einsele^1^, Leo Rasche^1,3^, K. Martin Kortüm^1^, Johannes M. Waldschmidt^1^

1 Department of Internal Medicine II, University Hospital of Würzburg, Würzburg, Germany

2 Department of Internal Medicine 5, Klinikum Nürnberg Nord, Nürnberg, Germany.

3 Mildred Scheel Early Career Center, Würzburg, Germany

**Corresponding author:**

Johannes M. Waldschmidt, MD

Department of Hematology and Oncology

University Hospital Würzburg

Oberdürrbacher Street 6

97080 Würzburg

E-Mail: waldschmid_j@ukw.de

**Supplementary Figures**


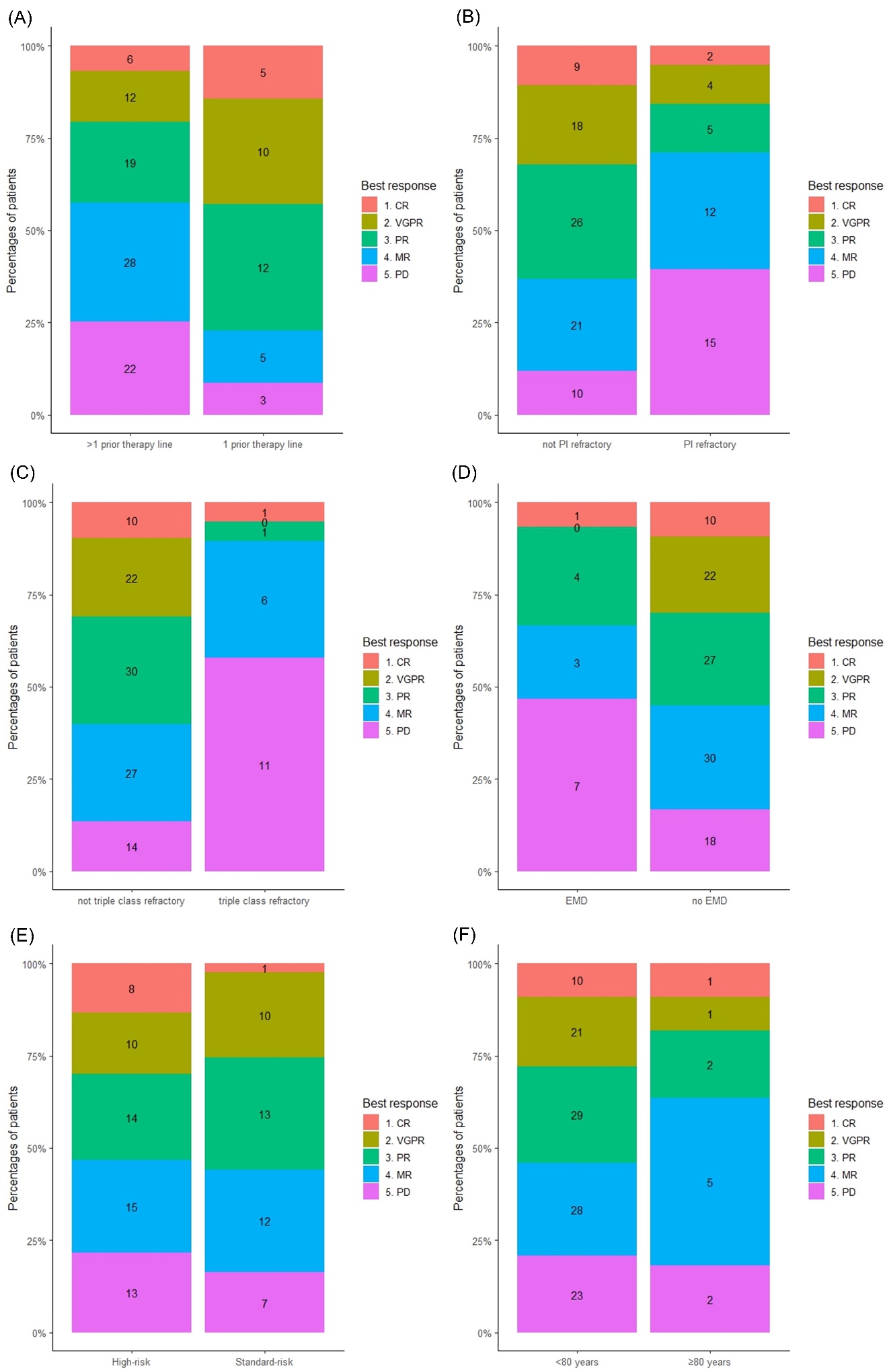


**Figure S1: Best response to ixazomib containing therapies in different subgroups: (A)** Patient who received ixazomib in the second line (one prior line of therapy) showed significantly higher ORR than those with >one prior lines of therapy (77.1% versus 42.5%, *P*=0.0006). **(B)** Patients being refractory to PI in previous therapy lines showed significantly lower ORR compared to the remaining patients (28.9% versus 63.1%, *P*=0.0008). **(C)** Triple class refractory patients demonstrated significantly inferior ORR than the others (10.5% versus 60.2%, *P*<0.0001). **(D-F)** EMD, high-risk cytogenetics and age of ≥80 years did not indicate lower ORR compared to other patients. CR - complete remission; EMD - extramedullary disease; MR - minor response; ORR - overall response rate; PD - progressive disease; PI - proteasome inhibitor; PR - partial remission; VGPR - very good partial remission.


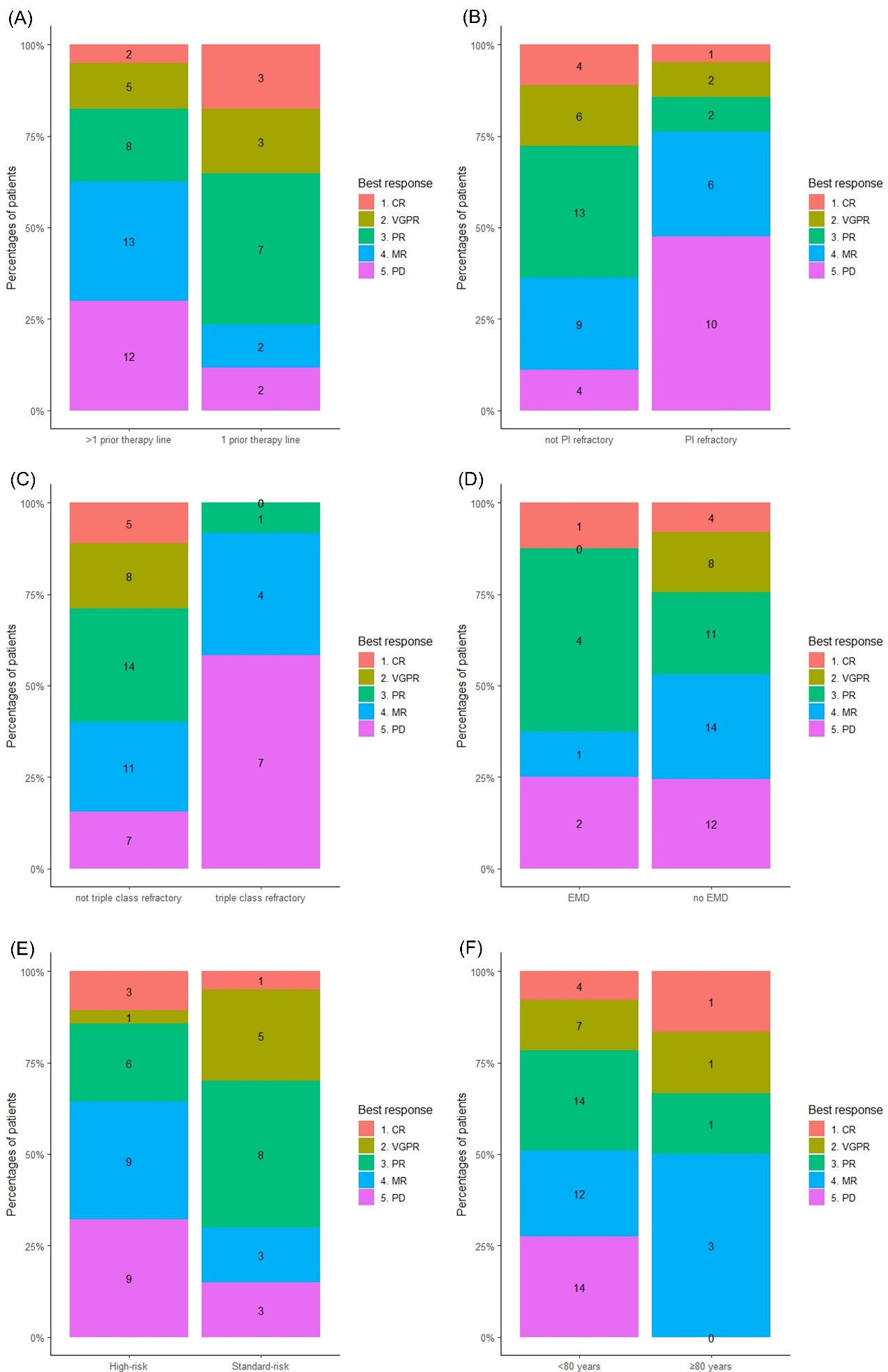


**Figure S2: Best response to Ixa-Rd in different subgroups: (A)** Patients who received Ixa-Rd in the second line (one prior line of therapy) showed significantly higher ORR than those with >one prior lines of therapy (76.5% versus 37.5%, *P*=0.0096). **(B)** Patients being refractory to PI in previous therapy lines showed significantly lower ORR compared to the remaining patients (23.8% versus 63.9%, *P*=0.0056). **(C)** Triple class refractory patients demonstrated significantly inferior ORR than the others (8.3% versus 60.0%, *P*=0.0011). **(D-F)** EMD and age of ≥80 years did not indicate lower ORR compared to other patients, but high-risk cytogenetics were associated with lower ORR compared with standard-cytogenetics in the Ixa-Rd subgroup (35.7% versus 70.0%, *P*=0.039). CR - complete remission; EMD - extramedullary disease; Ixa-Rd - ixazomib, lenalidomide and dexamethasone; MR - minor response; ORR - overall response rate; PD - progressive disease; PI - proteasome inhibitor; PR - partial remission; VGPR - very good partial remission.


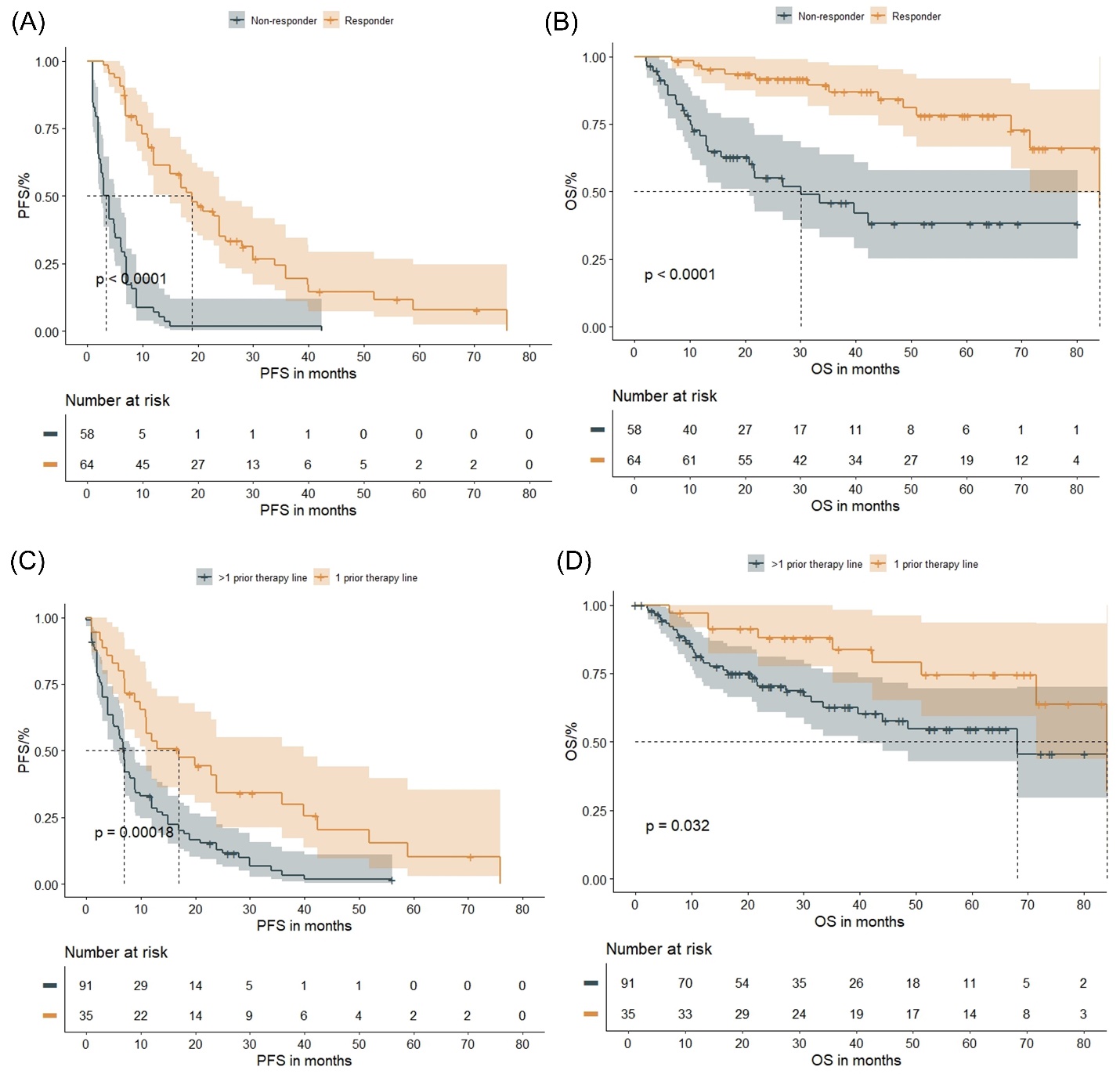


**Figure S3: Impact of response and prior lines of therapy on survival outcome in the entire group:** The figure demonstrate the PFS and OS in patients responding versus not responding to ixazomib (**A-B**), in patients with only 1 versus >1 prior therapy line (**C-D**). OS - overall survival; PFS - progression free survival; Log-rank *P* values are provided.


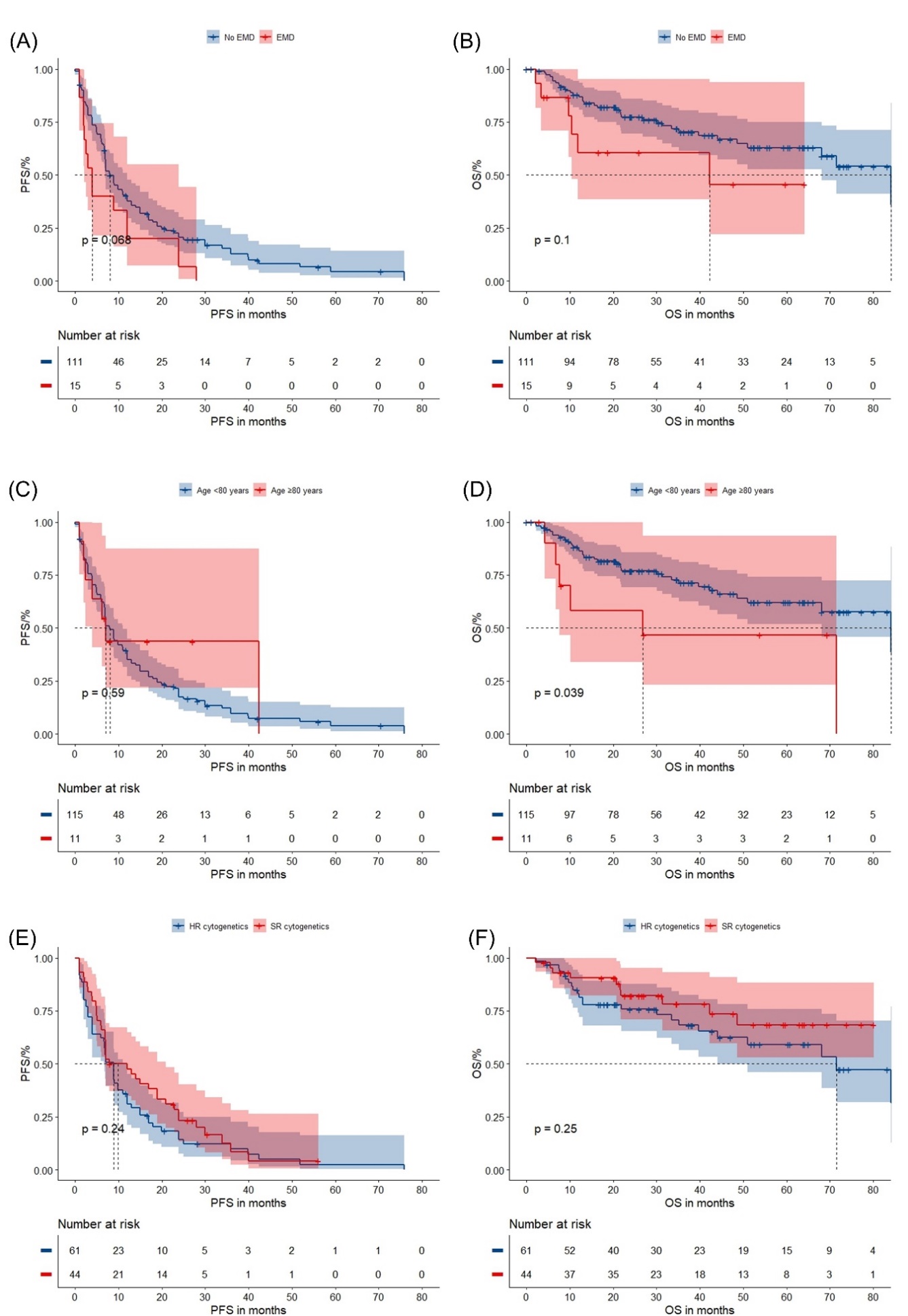


**Figure S4: Impact of EMD, age and cytogenetics on survival outcome in the entire group:** The figures demonstrate the PFS and OS in patients with versus without EMD (**A-B**), in patients aged <80 years versus ≥80 years (**C-D**), and in patients with HR versus SR cytogenetics (**E-F**). EMD - extramedullary disease; HR - high-risk; OS - overall survival; PFS - progression free survival; SR - standard-risk. Log-rank *P* values are provided


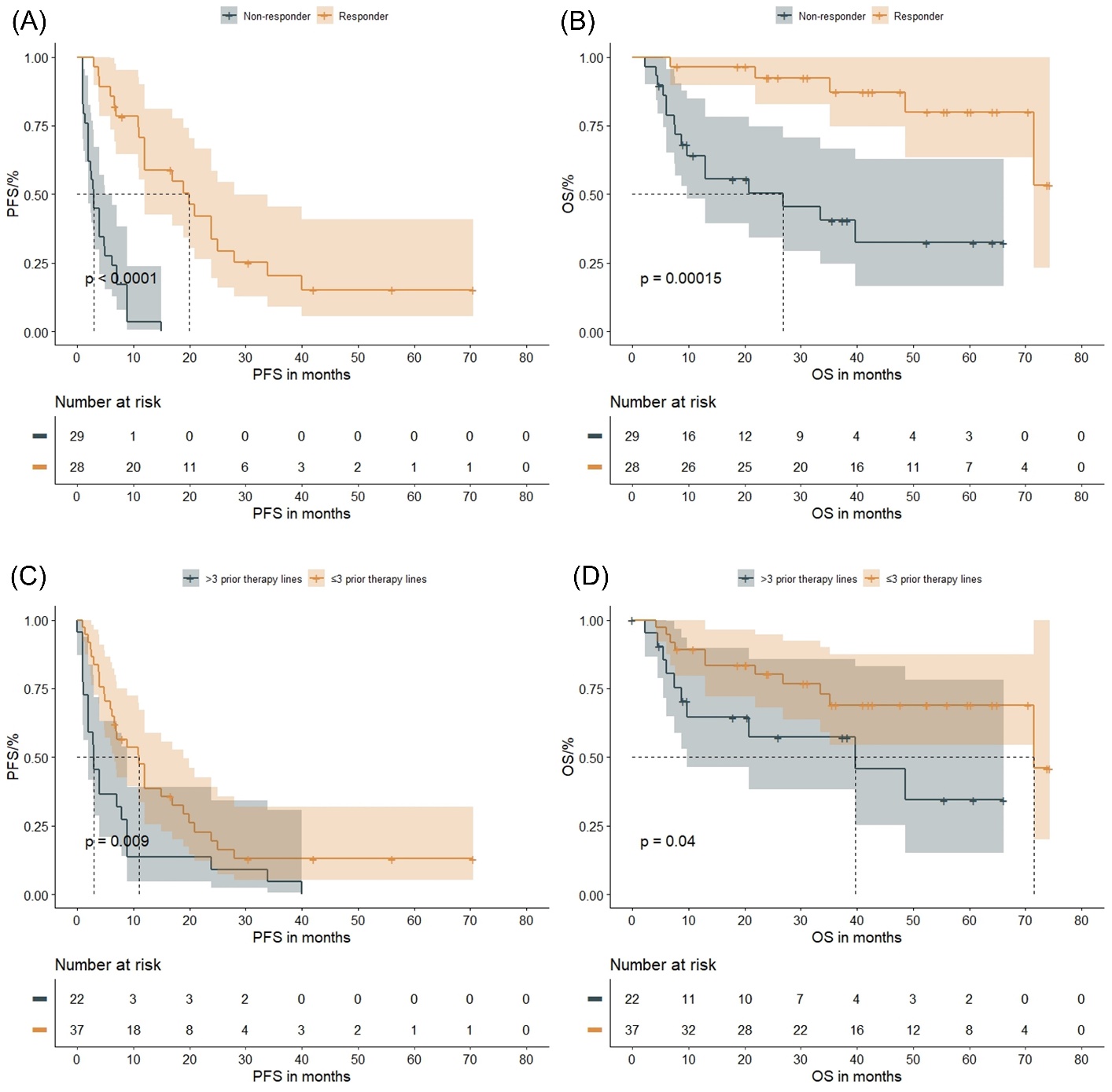


**Figure S5: Impact of response and prior lines of therapy on survival outcomes in the Ixa-Rd subgroup:** The figures demonstrate the PFS and OS in patients responding versus not responding to ixazomib (**A-B**), in patients with ≤3 versus >3 prior therapy line (**C-D**). OS - overall survival; PFS - progression free survival; Log-rank *P* values are provided.


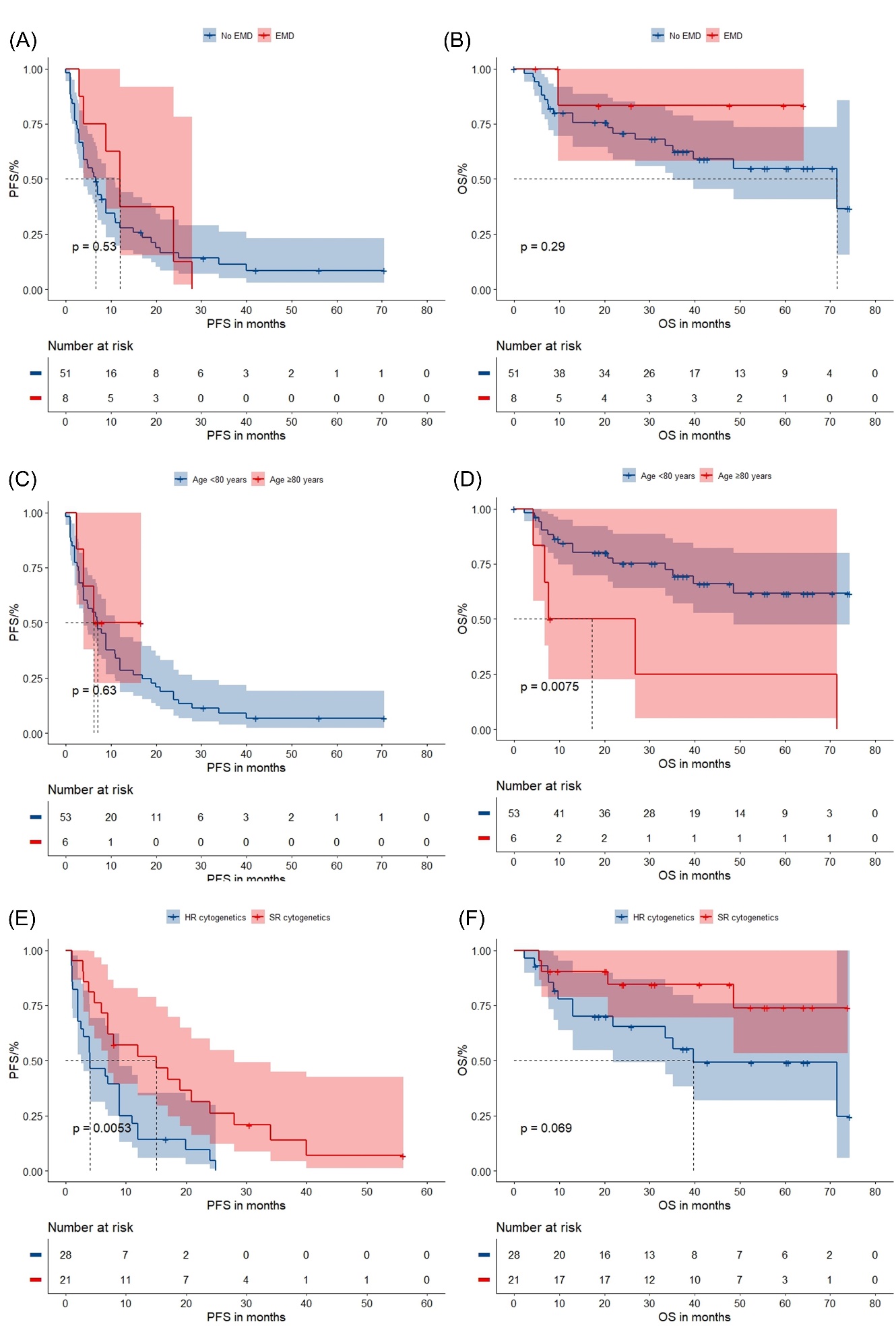
 **Figure S6: Impact of EMD, age and cytogenetics on survival outcome in the Ixa-Rd subgroup:** The figures demonstrate the PFS and OS in patients with versus without EMD (**A-B**), in patients aged <80 years versus ≥80 years (**C-D**), and in patients with HR versus SR cytogenetics (**E-F**). EMD - extramedullary disease; HR - high-risk; OS - overall survival; PFS - progression free survival; SR - standard-risk. Log-rank *P* values are provided.


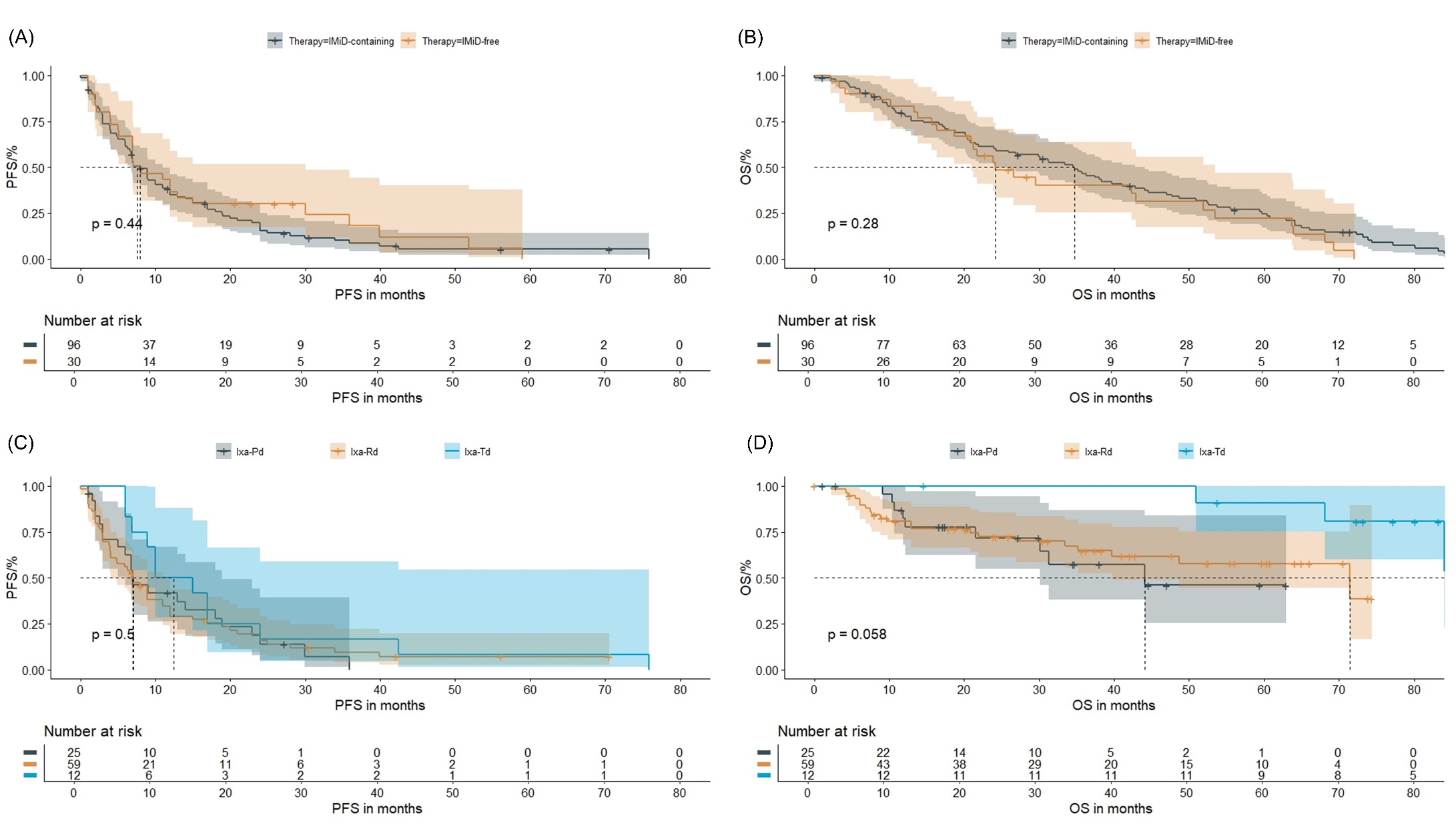


**Figure S7: IMiD-containing regimens survival outcomes:** The figures demonstrate the PFS and OS in patients treated with IMiD-containing versus IMiD-free combinations (**A-B**), and in patients treated with different IMiD-containing regimens (**C-D**). IMiD - immunomodulatory drugs; Ixa-Pd - ixazomib, pomalidomide, dexamethasone; Ixa-Rd - ixazomib, lenalidomide, dexamethasone; Ixa-Td - ixazomib, thalidomide, dexamethasone; OS - overall survival; PFS - progression free survival. Log-rank *P* values are provided.


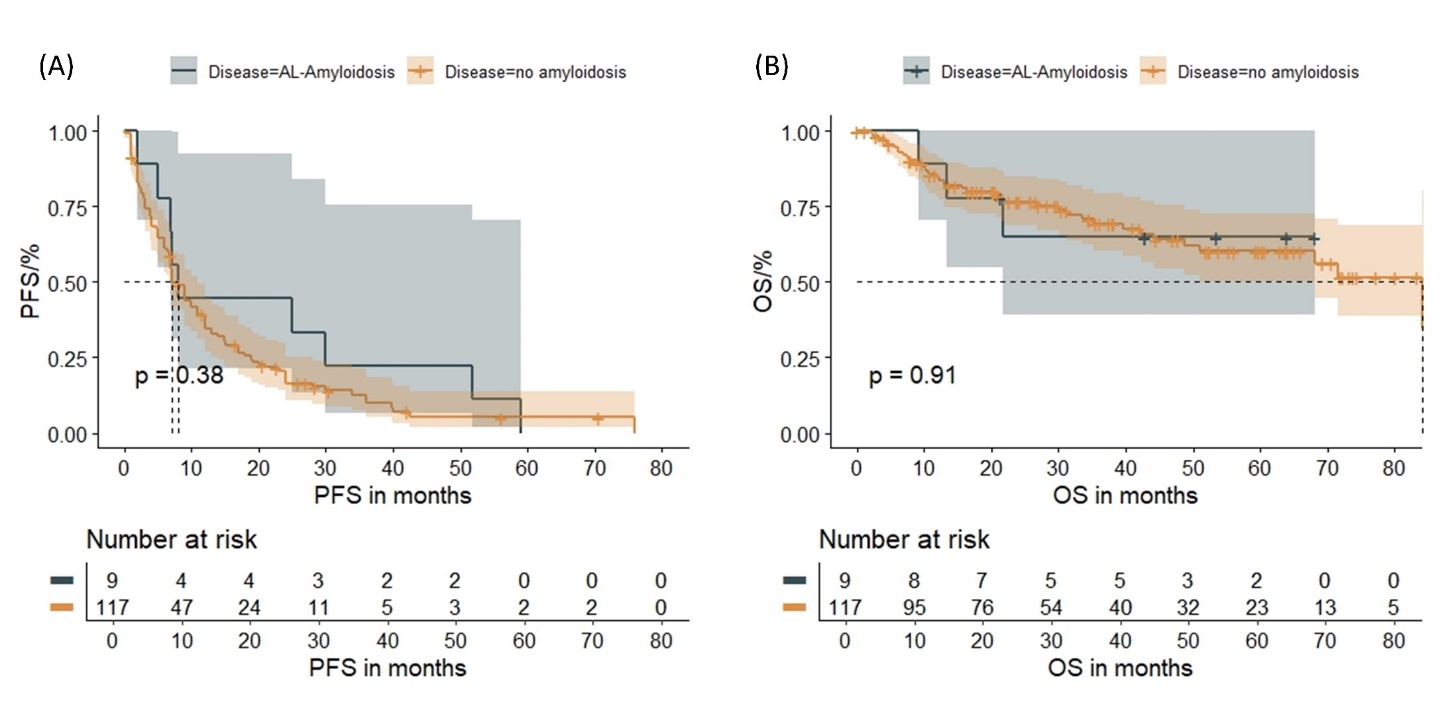


**Figure S8: The presence of AL amyloidosis and survival outcomes:** The figures demonstrate the PFS and OS in patients with versus without MM-associated AL amyloidosis (**A-B**). OS - overall survival; PFS - progression free survival. Log-rank *P* values are provided.


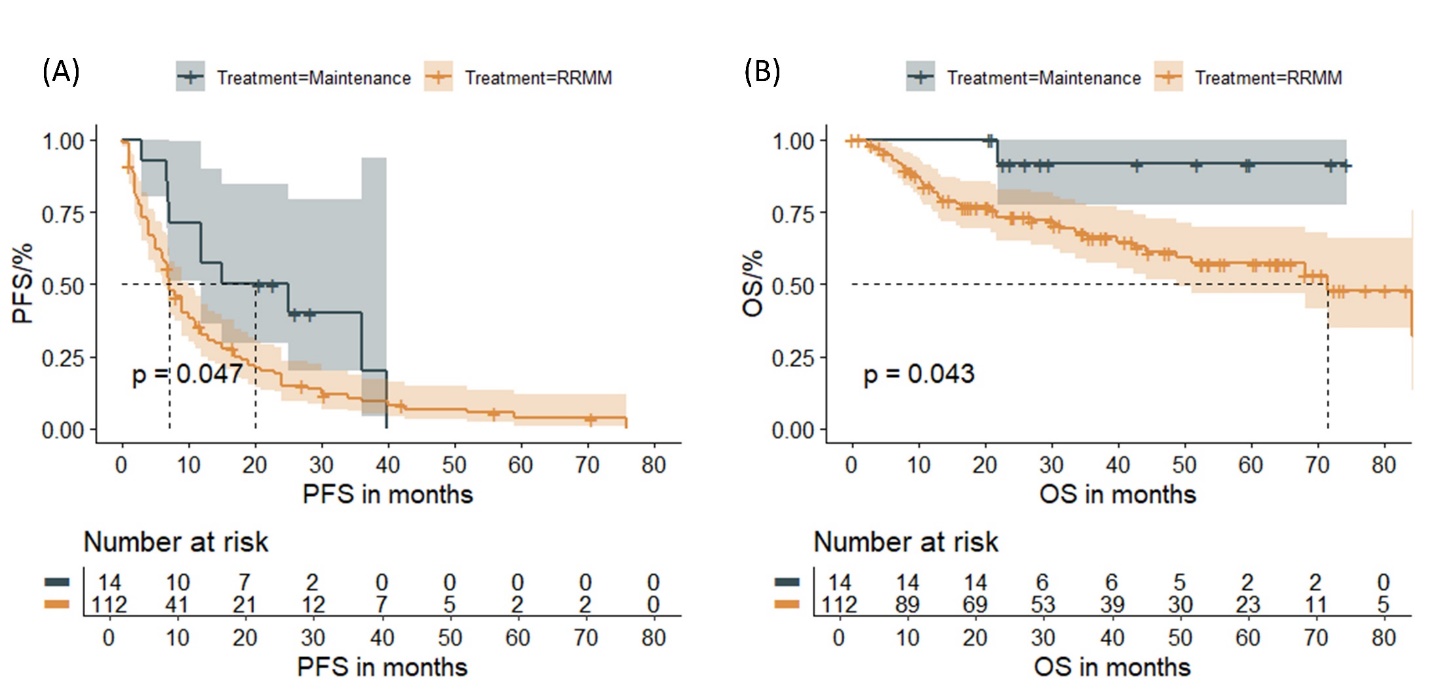


**Figure S9: Survival outcomes in patients treated with ixazomib-containing maintenance versus due to relapse or refractory disease:** The figures demonstrate the PFS and OS in patients treated with ixazomib-containing maintenance versus those who received ixazomib due to relapse or refractory disease (**A-B**). OS - overall survival; PFS - progression free survival. RRMM – relapsed/refractory multiple myeloma. Log-rank *P* values are provided.
